# Epidemiology of Wrestling Injuries in Female Athletes at US Emergency Departments

**DOI:** 10.7759/cureus.76096

**Published:** 2024-12-20

**Authors:** Jasmeet Khera, Nikhil Furtado, Sai Geetika Guturu, Jacob Lin, Matthew Dilisio

**Affiliations:** 1 Sports Medicine, Creighton University School of Medicine, Omaha, USA; 2 Internal Medicine, Creighton University School of Medicine, Omaha, USA; 3 Orthopedic Surgery, OrthoNebraska, Omaha, USA; 4 Orthopedic Surgery, Creighton University School of Medicine, Omaha, USA

**Keywords:** adolescent, emergency departments, female athletes, injuries, pediatric, sports

## Abstract

Purpose

Wrestling injuries in female athletes is an understudied topic. The aim of this study is to characterize the injuries that occur in female wrestlers aged five to 18 who presented to United States (US) emergency departments (ED) from 2014-2023 and compare patterns between youth and adolescent wrestlers.

Method

This was a retrospective comparative study including female patients aged five to 18 years of age presenting to the ED due to an injury related to their participation in the sport of wrestling. Data was extracted from the National Electronic Injury Surveillance System (NEISS) from 2014 to 2023. Information extracted included patient demographics, injury location, disposition, diagnosis as well as a narrative for each patient. Injury distribution for youth (five to 11) and adolescents (12-18) was evaluated using chi-squared.

Results

An estimated 28,824 female wrestlers (841 NEISS cases) presented to US EDs during the study period. The most frequently injured body parts were the head (16.5%), shoulder (14.0%), knee (11.1%), and elbow (10.7%). Strains/sprains were the most common injuries in both youth (27.9%) and adolescents (30.3%). Fractures were significantly more common among youth wrestlers (23.1%) compared to adolescents (13.6%, p < 0.05). The distribution of injured body parts was also significantly different between groups (p < 0.05). In youth wrestlers, the most common injury sites were the head (11.5%, 95% CI 20.0-57.7%), wrist (10.6%, 95% CI 9.85-47.4%), and elbow (9.6%, 95% CI 16.3-56.6%). For adolescents, the head (17.2%, 95% CI 24.9-34.9%), shoulder (14.9%, 95% CI 31.4-43.5%), and knee (11.7%, 95% CI 27.7-41.0%) were most frequently injured. Overall, more than 60% of injuries occurred above the waist in both groups.

Conclusion

Adolescent female wrestlers experienced more injuries compared to youth wrestlers, with most injuries occurring above the waist. Factors such as increased mat time, hormonal changes, and higher practice intensity may contribute to this disparity. Injury prevention strategies, including adequate mat spacing and rule enforcement during practices as well as upper body strength training, are recommended. Further research is needed to identify mechanisms and specific techniques linked to higher injury risks to enhance safety in female wrestling.

## Introduction

Women's wrestling is one of the fastest-growing sports in the United States. The sport now has over 50,000 participants at just the high school level in 2023 versus just 804 in 1994 [1,2], with 2023 being the year where women have their own officially established weight classes rather than having the same weight divisions as the men [3]. Wrestling has one of the highest injury rates at 2.5 per 1000 athlete exposures, second only to football, which was 4.36 per 1000 athlete exposures [4]. Unlike other sports, wrestling male and female wrestlers practice with each other and compete against one another from the youth level up to the high school level. Guidelines and regulations surrounding their participation are different, with men being allowed to be at a minimum of 7% body fat and women 12%, according to National Wrestling Coaching Association (NWCA) guidelines [5]. Regulations have been implemented to minimize injuries, as excessive weight-cutting has been shown to increase the risk of injury [6]. Youth wrestling does not have the same rules and regulations as the high school level, with many different community organizers running events and many private clubs operating within communities. 

Some studies examine differences in injuries and injury patterns between youth and adolescent wrestlers in males and differences in injuries and injury patterns between men and women [7,8]. Studies have demonstrated that female wrestlers at the elite and Olympic levels have lower observed injury rates than males [9], as well as that females are less likely to present with fractures but are more likely to present with sprains [7-9]. However, no studies have examined the differences in injuries and injury patterns between youth and adolescent female wrestlers. This is important to understand as the degree to which injuries occur and how severe they are between the two groups is currently unknown. Overall, youth athletes are more prone to avulsion fractures rather than ligament and muscle-tendon injuries [10]. Given this knowledge gap, we are setting out to compare patterns of injury between female wrestlers in youth and middle/high school levels and describe the injuries that occur across these age groups.

## Materials and methods

The Consumer Product Safety Commission’s National Electronic Injury Surveillance System (NEISS) is a publicly available database. The database collects information on consumer product-related injuries occurring in the US. This is then used to produce nationwide estimates of product-related injuries. The national estimate is based on a national representative probability sample in the US and its territories. Hospitals are stratified into five groups, with four representing EDs of various sizes and one representing EDs from children’s hospitals, with each hospital having a certain weight depending on the size of the hospital and the number of hospitals that size. National estimates are then calculated by summing all of the raw NEISS cases that presented to participating EDs.

The data was pulled from the NEISS for patients treated from Jan 1st, 2014 to December 31, 2023. This study will include all ED visits from wrestling-associated injuries. NEISS codes Wrestling (activity, Apparel, or Equipment Product code 1270). We will review the narrative of each record and exclude anything that is not derived from Folkstyle/Scholastic, Freestyle, or Greco-Roman. We will only pull data from females. 

The NEISS database collects information on patient treatment date, patient age, gender, sex, disposition, body part injured, locale of where the injury occurred, type of product involved in injury, alcohol or drug use, and a narrative describing the circumstances of the injury. Locales included in our study will be injuries that occurred at school or any other place of sports or recreation. All other locales, such as homes, farms/ranches, streets, stores, office buildings, restaurants, churches, hotels, motels, hospitals, nightclubs, theaters, and mobile homes, will be excluded. The age groupings for analysis will be five to 11 years and 12-18 years to approximate elementary school age and middle/high school age, as these groups practice and compete together. The mechanism of injury was characterized by manually going through the narratives and adding the weights to get the national estimate. Injuries sustained from the athlete falling were categorized as “fell”; injuries sustained from the athlete being hit were categorized as “hit.” Injuries that resulted from the athlete being thrown or twisting a body part were categorized as “thrown” or “twist,” respectively. Injuries that did not fit into those categories but had some type of specification, such as overexertion or soreness, were categorized as “other.” The not-specified category was used for injuries with no explanation in the narrative other than “wrestling.” All NEISS diagnoses are pulled and analyzed in RStudio (Version 2024.04.2+764) to describe the diagnosis, trends in injuries, and most commonly injured body parts. A Pearson chi-squared analysis assessed the differences between the two age groups. 

## Results

Characteristics of study subjects

**Figure 1:**
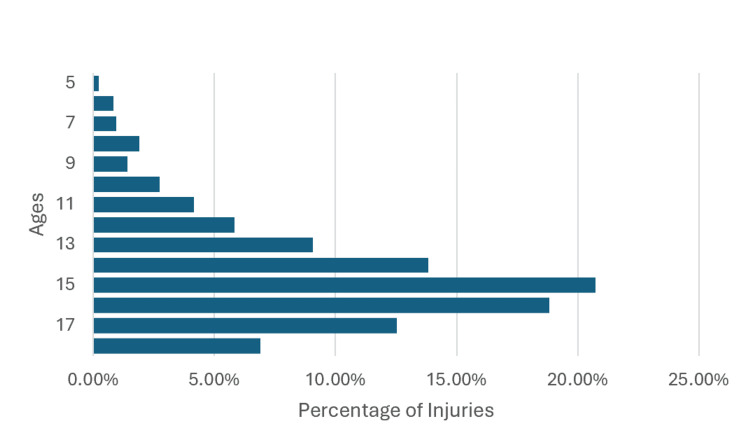
Percentage of total injuries in each age group

**Figure 2:**
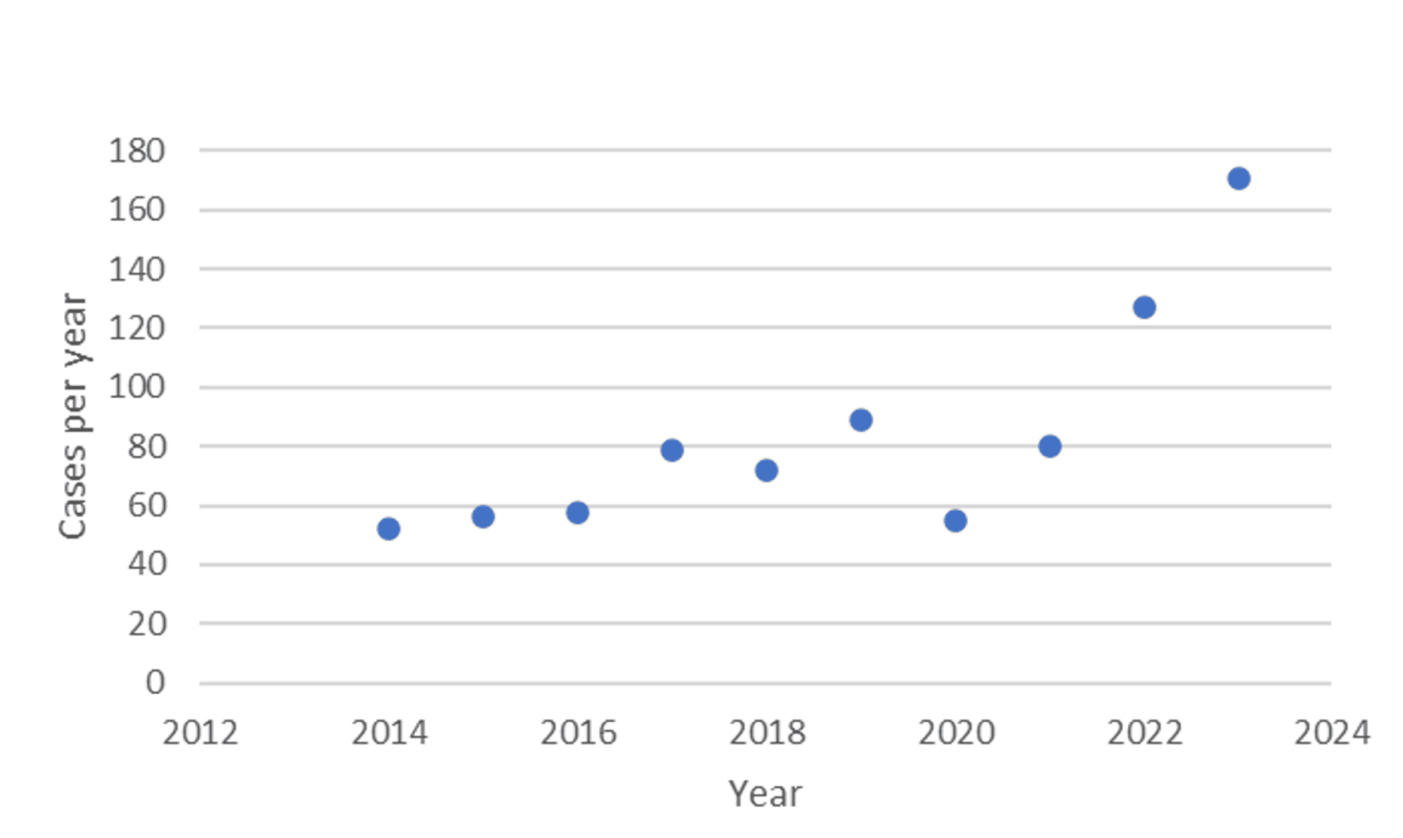
Number of National Electronic Injury Surveillance System (NEISS) cases per year

There were an estimated 28,823 (841 NEISS visits) ED visits for wrestling-related injuries in youth and adolescent female wrestlers aged five to 18 from January 2015 to December 2023. Among all injured female wrestlers, 88.9% of all visits occurred in the 12-18 age range. The five to 11 year age range accounted for only 11.1% of all visits. The age distributions of these visits and age ranges are depicted in Figure 1 and Figure 2, respectively. Of these injuries, 15-year-olds were the most injured, comprising 20.74% of all injuries. The next highest injured age was 16-year-olds, with 18.83% of all injuries.

Main results

Table 1 illustrates the characteristics of injuries stratified by body parts affected by female wrestling-related ED injury visits. The most commonly injured body part was the head, which constituted a national estimate of 16.53% of all injuries from NEISS Cases. The finger was the least injured body part, constituting only 3.92% of injuries by NEISS cases.

**Table 1:** Number of injuries per body part as reported by National Electronic Injury Surveillance System (NEISS) cases and National Estimates. Percentage is calculated based off of National Electronic Injury Surveillance System cases

Body Part	NEISS Cases	National Estimate	%
Head	139	4266	16.53
Shoulder	118	4275	14.03
Knee	93	3140	11.06
Elbow	90	3081	10.70
Ankle	66	2566	7.85
Upper Trunk	62	2434	7.37
Neck	40	1245	4.76
Lower Trunk	37	1573	4.40
Wrist	34	1216	4.04
Finger	33	1191	3.92
Head	23	4266	2.73

There were approximately seven times more injuries in the adolescent female wrestler age group (12-18) compared to the youth female wrestler age group (five to 11) over the 10-year period (see Table 2). The distribution of injury diagnoses was significantly different (p=2.2e-16) in youth and adolescent age groups, with the most common injury being a strain/sprain, accounting for 27.9% and 30.3% of all injury diagnoses, respectively. The next most common injury was another (24.0% and 22.4%, respectively), followed by fracture (23.1% and 13.6%, respectively). However, the fourth most common injury for the adolescent group was a concussion, accounting for 11.3% of injuries. In contrast, concussions were the second least common in the youth group, accounting for 4.8% of injuries. The distribution of body parts injured by location was significantly different among the two groups (p=2.2e-16). In the youth group, the head was the most common location (11.5%, 95% CL 20.0-57.7%), followed by the wrist (10.6%, 95% CL 9.85-47.4%) and elbow (9.6%, 95% CL 16.3-56.6%). The most common injury location in the adolescent group was the head (17.2%, 95% CL 24.9-34.9%), however the next most common was the shoulder (14.9%, 95% CL 31.4-43.5%), followed by the knee (11.7%, 95% CL 27.7-41.0%). In both groups, most injuries occurred above the waist (>60% of all injuries).

**Table 2:** Injuries stratified by diagnosis using National Electronic Injury Surveillance System cases 1: p is equal to 2.2e-16 2: p is equal to 2.2e-16

	Age 5–11		Age 12–18	
Characteristic	n	% (95% CL)	n	% (95% CL)
Total Injuries	104	--	737	--
Injuries by Diagnosis^1^				
Strain/ Sprain	29	27.9 (26.7-49.3)	223	30.3 (36.1–44.8)
Other	25	24.0 (10.9-29.5)	165	22.4 (30.6–40.5)
Fracture	24	23.1 (12.6-34.8)	100	13.6 (27.0–39.2)
Concussion	5	4.8 (14.6-83.5)	83	11.3 (23.7–36.3)
Contusion, Abrasion	12	11.5 (19.1-60.9)	75	10.2 (25.1–38.7)
Internal Injury	6	5.8 (11.5-59.6)	38	5.2 (18.5–35.6)
Dislocation	1	1.0 (NA)	39	5.3 (21.8–38.7)
Injuries by Location^2^				
Head	12	11.5 (20.0–57.7)	127	17.2 (24.9–34.9)
Shoulder	8	7.7 (4.14–35.4)	110	14.9 (31.4–43.5)
Knee	7	6.7 (2.27–51.7)	86	11.7 (27.7–41.0)
Elbow	10	9.6 (16.3–56.6)	80	10.9 (27.8–40.2)
Ankle	6	5.8 (12.0–55.3)	60	8.1 (30.9–47.9)
Lower Arm	9	8.7 (0.575–32.3)	11	1.5 (10.7–50.8)
Neck	5	4.8 (4.81–15.5)	35	4.7 (23.0–45.2)
Lower Trunk	4	3.8 (6.17–79.4)	33	4.5 (30.6–54.3)
Wrist	11	10.6 (9.85–47.4)	23	3.1 (25.1–53.1)
Finger	7	6.7 (9.41–59.7)	26	3.5 (23.9–49.1)

Excluding cases where a primary mechanism of injury could not be identified, the most common primary mechanism of wrestling injury was falling, constituting 91 of the NEISS cases. The next most common mechanism was hit, which comprised 85 NEISS cases. The national estimate revealed that hit was the most common mechanism, accounting for 8.67% of all injuries, followed by fall, which accounted for 7.92% of all injuries (Table 3).

**Table 3:** Injury Incidence characterized by Primary Mechanism of Injury Note, “Not specified” indicates that a primary mechanism other than engaging in wrestling was unable to be determined from the case narrative.

Mechanism of Injury	NEISS Cases	National Estimate	%
Fell	91	2284	7.92%
Hit	85	2469	8.57%
Twist	47	1634	5.67%
Thrown	38	1462	5.1%
Other	20	550	1.9%
Not Specified*	518	18667	64.76%

The distribution of injuries by race was similar in the two groups (Table 4). The most common race injured in both the youth and adolescent age groups was White, comprising 51.0% and 47.6%, respectively. The next most common race, excluding the cases of non-specific reported race, was Black, which constituted 11.5% and 11.1%, respectively.

**Table 4:** Characteristics of different races

Characteristic	n Age 5-11	% (95% CL)	n Age 12-18	% (95% CL)
Not specified (N.S.)	26	25.0 (23.9-47.6)	221	30.0 (30.4-38.5)
White	53	51.0 (25.9–43.2)	351	47.6 (34.4-41.2)
Black	12	11.5 (4.8-26.3)	82	11.1 (14.7-25.6)
Other	10	9.6 (2.9–41.8)	65	8.8 (27.2-42.7)
Asian	2	1.9 (14.9–18.4)	13	1.8 (28.1-63.7)
American Indian/ Alaska Native	0	NA	1	NA
Native Hawaiian/ Pacific Islander	1	NA	4	0.5 (8.7-59.5)

## Discussion

Prior studies on females have not been done due to a lack of data from low participation rates amongst females in wrestling. Studies done in male participants have shown similar patterns of injuries to our study [5], with strains/sprains being the most common injury. However, unlike our study, males had no significant difference in the injuries by diagnosis. The injuries by location were not the same as well. In males, the wrist/hand/finger was the most commonly injured body part, whereas in our study, the head was the most common. Our study also found that the wrist and finger were two of the least common injuries in females. Furthermore, studies on college male athletes found the elbow and hand were the most commonly injured body parts, with lacerations being the highest proportion of diagnoses [11].

Older wrestlers (12-18 years) were found to have about seven times greater injuries than youth wrestlers (five to 11). Previous studies have shown similar results [12]. This could be due to a variety of factors. Youth wrestlers tend to be more flexible and have seasons that are shorter in duration compared to older wrestlers. Wrestling matches are also longer once wrestlers start competing in high school, leading to more mat time and thus increasing the likelihood of them getting injured. Older wrestlers also have longer seasons compared to youth wrestlers. Older wrestlers also tend to be stronger, which could contribute to injury discrepancies. Older wrestlers could also have more size and strength discrepancies within their respective weight classes than youth wrestlers, which could contribute to higher incidents resulting in injury. Further study into wrestler’s opponents, as well as the context (i.e., practice, competition) of the incident, would provide further insight into the factors contributing to injury rates of adolescent female wrestlers.

Younger wrestlers (five to 11 years) experienced higher fracture rates than adolescent wrestlers (12-18 years), 23.1% and 13.6%, respectively. Failure to know proper fall techniques could contribute to the higher rates of fractures among youth wrestlers, particularly at the shoulder, elbow, arm, wrist, and finger. Further study into the distribution of fracture locations and causes would guide improved education on fall techniques. Older wrestlers had higher rates of dislocation than youth wrestlers, 5.3% and 1%, respectively, as well as higher rates of concussions than youth wrestlers, 11.3% and 4.8%, respectively. Older wrestlers tend to be stronger and faster, which could contribute to higher forces in the mechanism of injuries. Older wrestlers also tend to participate at more competitive levels, which can lead to more aggressive matches, with athletes willing to put their head in potentially compromising positions, particularly in the longer matches as they fatigue. Further study of the incidents leading to concussions in youth and adolescent wrestlers could point towards the need for increased emphasis on safe head positioning.

Compared to other sports, the types of injuries are similar [13-15]. Girls’ basketball and soccer reported head/face/neck injuries being the most common during competition [14], similar to wrestling. However, our study shows a higher representation of fractures than other sports. This could be due to many factors. Our study only looks at participants who presented to the ED, thus overrepresenting patients with higher acuity problems. Other studies included whether injuries were from practice or competition and classified injuries as acute vs. chronic [13,14], thus making fractures less represented than if the studies had looked at just acute injuries or injuries presenting to the ED. Women’s wrestling is also unique compared to other sports because it is a sport where both boys and girls compete against each other in practice and competition. This does not occur regularly in sports such as basketball and soccer. Historically, due to the low number of girls in wrestling, they have often had to join the boys’ team. As stated in the background section, girls did not have their own weight classes until 2023. Girls will have more opportunities to wrestle with other girls as the sport grows. A robust recruitment effort is suggested to get more girls into wrestling, potentially resulting in a safer environment for girls to practice and compete in. 

There was a significant difference when comparing body parts injured. Youth wrestlers had a higher proportion of wrist and elbow injuries, whereas older wrestlers had a higher proportion of shoulder and knee injuries. Various factors could contribute to this, the most significant of which could be hormonal differences between the two age groups. Older athletes are at an age where they are starting to have menstrual periods, with many young athletes also starting to take birth control [16]. Studies have shown that the ovulatory phase of menstruation is associated with an increased risk of injury due to changes in laxity and strength [17].

Limitations

The most common injury type was not specified, i.e., the narrative did not provide any further explanation other than that the patient was wrestling. Other studies have shown that most injuries occur during hard wrestling, with the most common wrestling situation resulting in an injury being the takedown [18]. The takedown could lead to several mechanisms of injury, such as falling if release from the takedown was not controlled or if the takedown itself resulted in a fall. Being hit by a takedown could also result in injury and being thrown using a takedown technique. Twisting could also result in injury from a takedown, depending on the takedown used. Current wrestling moves also can contribute to these mechanisms. For example, there is a move in wrestling called the “twister,” where you position yourself on top of the opponent in a way that lets you apply torsional pressure through their spine. Other moves such as the half nelson, armbar, turks, and stacks can result in twisting injuries and injuries directly to the shoulder. It is unclear as to what movements resulted in what injuries. Getting this information would help guide suggestions on preventative strategies and reconsidering acceptable moves. In competition, these moves are very regulated because the referee is watching and can call a “potentially dangerous” call, whereas this may not be the case in practices where a coach must watch multiple wrestlers simultaneously, thus missing it. Mechanisms such as falls could potentially be prevented by providing adequate mat space during practices, having only a certain number of people wrestling during a period of time while their teammates and coaches surveil the space could work to decrease these types of injuries.

While the ED is a good location to gather data on injury nationally, future national research could be done closer to competition or practices. This would allow for a more accurate representation of wrestling injuries since many injuries may not require a visit to the ED. Thus, just looking at ED visits could be an underrepresentation of injuries in these athletes since athletes with minor injuries will not come to the ED. Also, additional studies would benefit from a more standardized and complete classification of injury mechanisms, body parts involved, and diagnoses. This would allow for better characterization of mechanisms of injuries as well as more specific diagnosis. With a more detailed characterization of diagnoses, more studies could be done regarding the type of follow-up care these athletes receive. For example, how many get surgeries for their injuries, go through physical therapy, take time off the sport, etc. Our study is also a retrospective study observational study which is a limitation. 

The age group in our study is meant to approximate the youth wrestling population to the adolescent wrestling population. This is because these are the groups that tend to wrestle in competition against each other and practice with each other. There is a potential overlap in these groups, which may confound our data. Our study also looks at wrestling injuries due to certain styles of wrestling, such as freestyle, folkstyle, and Greco-Roman. This was done by looking through the narrative and eliminating anything such as sumo wrestling, mud wrestling, or any other form outside the targeted styles. Despite this, some cases may have been erroneously included or excluded. 

## Conclusions

Injuries in female wrestlers have gone up amongst youth and adolescent athletes. There is a significant difference in the distribution of injuries between the two groups, but there is a similar diagnosis pattern. Most injuries occur above the waist. Factors such as increased mat time, hormonal changes, and higher practice intensity may contribute to this disparity. Injury prevention strategies are recommended, including adequate mat spacing and rule enforcement during practices and increased recruitment into the sport. Further research is needed to identify mechanisms and specific techniques linked to higher injury risks to enhance safety in female wrestling.
